# Supplementary material for: New Onset and Exacerbation of Autoimmune Bullous Dermatosis Following COVID-19 Vaccination: A Systematic Review
Source: Vaccines (Basel). 2024 Apr 26;12(5):465. doi: 10.3390/vaccines12050465 (PMC11125893; doi:10.3390/vaccines12050465)
Supplement: Supplementary file 1 [file vaccines-12-00465-s001.zip › vaccines-2923635-supplementary.pdf]

Table S1. Search strategy.

Database	Final syntax
CENTRAL (Cochrane Library)	#1 MeSH descriptor: [COVID-19 Vaccines] explode all trees<br>#2 (COVID-19):ti,ab,kw<br>#3 (SARS-CoV-2):ti,ab,kw<br>#4 (vaccine*):ti,ab,kw<br>#5 (COVID-19) or (COVID-19 vaccin*) or (COVID-19 virus vaccin*) or (SARS-CoV-2) or (SARS-CoV-2 vaccin*)<br>#6 (ChAdOx1 nCoV-19):ti,ab,kw<br>#7 (Ad26COVS1):ti,ab,kw<br>#8 (BBIBP-CorV):ti,ab,kw<br>#9 (CoronaVac):ti,ab,kw<br>#10 Covaxin):ti,ab,kw<br>#11 (KoviVac):ti,ab,kw<br>#12 (COVI-VAC):ti,ab,kw<br>#13 (RBD-Dimer):ti,ab,kw<br>#14 (ZF2001):ti,ab,kw<br>#15 (NVX-CoV2373):ti,ab,kw<br>#16 (EpiVacCorona):ti,ab,kw<br>#17 (Medicago Plant-based VLP):ti,ab,kw<br>#18 (Covishield):ti,ab,kw<br>#19 (Sputnik V, Sputnik Light):ti,ab,kw<br>#20 (mRNA-1273):ti,ab,kw<br>#21 (BNT162b2):ti,ab,kw<br>#22 (ZyCoV-D):ti,ab,kw<br>#23 #1 OR #2 OR #3 OR #4 OR #5 OR #6 OR #7 OR #8 OR #9 OR #10 OR #11 OR #12 OR #13 OR #14 OR #15 OR #16 OR #17 OR #18 OR #19 OR #20 OR #21 OR #22<br>#24 MeSH descriptor: [Skin Diseases, Vesiculobullous] explode all trees<br>#25 MeSH descriptor: [Pemphigoid, Bullous] explode all trees<br>#26 MeSH descriptor: [Pemphigus] explode all trees<br>#27 MeSH descriptor: [Linear IgA Bullous Dermatosis] this term only<br>#28 (bullous dermatos*):ti,ab,kw<br>#29 (bullous pemphigoid):ti,ab,kw<br>#30 (pemphigoid gestationis):ti,ab,kw<br>#31 (dystonin):ti,ab,kw<br>#32 (linear IgA bullous dermatos*):ti,ab,kw<br>#33 (pemphigus foliaceus):ti,ab,kw<br>#34 (pemphigus vulgaris):ti,ab,kw<br>#35 (pemphigus vegetan*):ti,ab,kw<br>#36 MeSH descriptor: [Desmoglein 1] this term only<br>#37 MeSH descriptor: [Desmoglein 3] this term only

	#38	(desmoglein 1):ti,ab,kw
	#39	(desmoglein 3):ti,ab,kw
	#40	#24 OR #25 OR #26 OR #27 OR #28 OR #29 OR #30 OR #31 OR #32 OR #33 OR #34 OR #35 OR #36 OR #37 OR #38 OR #39
	#41	#23 AND #40
MEDLINE	1.	exp COVID-19 Vaccines/
	2.	covid-19 vaccines.mp.
	3.	exp SARS-CoV-2 Vaccines/
	4.	SARS-CoV-2 vaccines.mp.
	5.	exp Coronavirus Disease 2019 Vaccines/
	6.	Coronavirus Disease 2019 vaccines.mp.
	7.	exp ChAdOx1 nCoV-19/
	8.	ChAdOx1 nCoV-19.mp.
	9.	exp Ad26COVS1/
	10.	Ad26COVS1.mp.
	11.	BBIBP-CorV.mp.
	12.	CoronaVac.mp.
	13.	Covaxin.mp.
	14.	KoviVac.mp.
	15.	DelNS1-nCoV-RBD LAIV.mp.
	16.	MV-014-212.mp.
	17.	COVI-VAC.mp.
	18.	RBD-Dimer.mp.
	19.	ZF2001.mp.
	20.	NVX-CoV2373.mp.
	21.	EpiVacCorona.mp.
	22.	Medicago Plant-based VLP.mp.
	23.	Ad5-nCoV.mp.
	24.	exp Covishield/
	25.	Covishield.mp.
	26.	Sputnik V.mp.
	27.	Sputnik Light.mp.
	28.	exp mRNA-1273/
	29.	mRNA-1273.mp.
	30.	BNT162b2.mp.
	31.	ZyCoV-D.mp.
	32.	or/1-31
	33.	exp Bullous Pemphigoid/
	34.	Bullous Pemphigoid.mp.
	35.	exp Pemphigoid/
	36.	Pemphigoid.mp.
	37.	exp Pemphigoid gestationis/

	38. Pemphigoid gestationis.mp.<br>39. exp Dystonin/<br>40. Dystonin.mp.<br>41. exp Pemphigoid, benign mucous membrane/<br>42. Pemphigoid, benign mucous membrane.mp.<br>43. exp Linear IgA Bullous Dermatosis/<br>44. exp Drug-induced Linear IgA Bullous Dermatosis/<br>45. Drug-induced Linear IgA Bullous Dermatosis.mp.<br>46. exp Chronic bullous disease of childhood/<br>47. Chronic bullous disease of childhood.mp.<br>48. exp Pemphigus/<br>49. Pemphigus.mp.<br>50. exp Pemphigus Vulgaris/<br>51. Pemphigus Vulgaris.mp.<br>52. exp Pemphigus Foliaceus/<br>53. Pemphigus Foliaceus.mp.<br>54. Paraneoplastic Pemphigus.mp.<br>55. Pemphigus Erythematosus.mp.<br>56. exp Desmoglein 1/<br>57. Desmoglein.mp.<br>58. exp Desmoglein 3/<br>59. Desmoglein 3.mp.<br>60. or/33-59<br>61. 32 and 60
Embase	#1 'sars-cov-2 vaccine'/exp OR 'sars-cov-2 vaccine'<br>#2 'vaxzevria'/exp OR vaxzevria'<br>#3 'ad26.cov2.s vaccine'/exp OR 'ad26.cov2.s vaccine'<br>#4 'covilo'/exp OR covilo<br>#5 'coronavac'/exp OR coronavac<br>#6 ‘covaxin’/exp OR covaxin<br>#7 ‘kovivac’/exp OR kovivac<br>#8 'delns1-ncov-rbd laiv'<br>#9 'mv 014 212'<br>#10 ‘covi-vac’/exp OR covi-vac<br>#11 'rbd dimer'<br>#12 ‘zifivax’/exp OR zifivax<br>#13 ‘nvx-cov2373 vaccine’/exp OR 'nvx-cov2373 vaccine'<br>#14 ‘epivaccorona’/exp OR epivaccorona<br>#15 medicago AND plant AND based AND vlp AND 'virus like particle vaccine'<br>#16 'ad5 ncov'/exp OR 'ad5 ncov'<br>#17 ‘vaxzevria’/exp OR vaxzevria

#18	'sputnik v vaccine'/exp OR 'sputnik v vaccine' OR 'sputnik light (vaccine)'/exp OR 'sputnik light (vaccine)'
#19	'elasomeran'/exp OR elasomeran
#20	'tozinameran'/exp OR tozinameran
#21	'zycov d'/exp OR 'zycov d'
#22	#1 or #2 or #3 or #4 or #5 or #6 or #7 or #8 or #9 or #10 or #11 or #12 or #13 or #14 or #15 or #16 or #17 or #18 or #19 or #20 or #21
#23	'pemphigoid'/exp OR pemphigoid
#24	'bullous pemphigoid'/exp OR 'bullous pemphigoid'
#25	'mucous membrane pemphigoid'/exp OR 'mucous membrane pemphigoid'
#26	'pemphigoid gestationis'/exp OR 'pemphigoid gestationis'
#27	'pemphigoid vegetans'/exp OR 'pemphigoid vegetans'
#28	'pemphigoid antibody'/exp OR 'pemphigoid antibody' OR 'pemphigoid antigen'/exp OR 'pemphigoid antigen' OR 'bp180 antibody' OR 'bp180 autoantibody' OR 'bp180 antigen' OR 'bp180 autoantigen'
#29	'bullous pemphigoid antigen 180' OR 'bullous pemphigoid antigen 1'/exp OR 'bullous pemphigoid antigen 1' OR 'bullous pemphigoid antigen 2'/exp OR 'bullous pemphigoid antigen 2'
#30	'linear iga bullous dermatosis'/exp OR 'linear iga bullous dermatosis'
#31	'chronic bullous disease of childhood'/exp OR 'chronic bullous disease of childhood''
#32	'adult linear iga disease'/exp OR 'adult linear iga disease'
#33	'pemphigus'/exp OR pemphigus
#34	'pemphigus vulgaris'/exp OR 'pemphigus vulgaris'
#35	'pemphigus foliaceus'/exp OR 'pemphigus foliaceus'
#36	'desmoglein 1'/exp OR 'desmoglein 1'
#37	'desmoglein 3'/exp OR 'desmoglein 3'
#38	'paraneoplastic pemphigus'/exp OR 'paraneoplastic pemphigus'
#39	'erythematous pemphigus'/exp OR 'erythematous pemphigus'
#40	#23 or #24 or #25 or #26 or #27 or #28 or #29 or #30 or #31 or #32 or #33 or #34 or #35 or #36 or #37 or #38 or #39
#41	#22 and #40

Table S2. Quality assessment of case reports.

Study	Q1	Q2	Q3	Q4	Q5	Q6	Q7	Q8	Overall
				**BP**					
Damiani 2021	Y	Y	Y	N	N	N	Y	Y	5
Khalid 2021	Y	Y	Y	Y	Y	N	Y	Y	7
Larson 2021	Y	Y	Y	Y	Y	N	Y	Y	7
Nakamura 2021	Y	Y	Y	Y	N	N	Y	Y	6
Pérez-López 2021	Y	Y	Y	N	Y	N	Y	Y	6
Tomayko 2021	Y	Y	Y	N	Y	N	Y	Y	6
Afacan 2022	Y	Y	Y	N	N	N	Y	Y	5
Agharbi 2022 (1)	Y	Y	Y	Y	N	N	Y	Y	6
Alshammari 2022	Y	Y	Y	N	N	N	Y	Y	5
Avallone 2022 (1)	Y	Y	Y	Y	N	N	Y	Y	6
Bailly-Caille 2022	Y	Y	Y	N	Y	N	Y	Y	6
Bardazzi 2022	Y	Y	Y	N	N	N	Y	Y	5
Bostan 2022	Y	Y	Y	N	N	N	Y	Y	5
Coto-Segura 2022	Y	Y	Y	N	N	N	Y	Y	5
Daines 2022	Y	Y	Y	Y	N	N	Y	Y	6
Dell'Antonia 2022	Y	Y	Y	Y	Y	N	Y	Y	7
Desai 2022	Y	Y	Y	Y	Y	N	Y	Y	7
Fu 2022	Y	Y	Y	N	N	N	Y	Y	5
Gambichler 2022	Y	Y	Y	Y	Y	N	Y	Y	7
Guo 2022	Y	Y	Y	Y	N	N	Y	Y	6
Hali (1) 2022	Y	Y	Y	Y	Y	N	Y	Y	7
Happaerts 2022	Y	Y	Y	N	N	N	Y	Y	5
Hung 2022	Y	Y	Y	N	N	N	Y	Y	5
Juay 2022	Y	Y	Y	Y	N	N	Y	Y	6
Maronese 2022 (1)	Y	Y	Y	N	N	N	Y	Y	5
Maronese 2022 (2)	Y	Y	Y	N	N	N	Y	Y	5
Nakahara 2022	Y	Y	Y	N	N	N	Y	Y	5
Nida 2022	Y	Y	Y	N	N	N	Y	Y	5
Pauluzzi 2022	Y	Y	Y	Y	N	N	Y	Y	6
Russo 2022	Y	Y	Y	N	N	N	Y	Y	5
Savoldy 2022	Y	Y	Y	N	Y	N	Y	Y	6
Schmidt 2022	Y	Y	Y	N	Y	N	Y	Y	6
Shakoei 2022	Y	Y	Y	Y	N	N	Y	Y	6
Shanshal 2022	Y	Y	Y	Y	Y	N	Y	Y	7
Wan 2022	Y	Y	Y	Y	Y	N	Y	Y	7
Young 2022	Y	Y	Y	Y	Y	N	Y	Y	7
Zhang 2022	Y	Y	Y	N	N	N	Y	Y	5

Baffa 2023	Y	Y	Y	Y	N	N	Y	Y	6
Dawoud 2023	Y	Y	Y	N	N	N	Y	Y	5
Hsieh 2023	Y	Y	Y	Y	N	N	Y	Y	6
Mulianto 2023	Y	Y	Y	N	N	N	Y	Y	5
Sun 2023	Y	Y	Y	Y	N	N	Y	Y	6
Üstün 2023	Y	Y	Y	Y	N	N	Y	Y	6
Yamamoto 2024	Y	Y	Y	N	Y	N	Y	Y	6
**PGes**									
Mustin 2023	Y	Y	Y	N	N	N	Y	Y	5
**MMP**									
Rungraungrayabkul 2023	Y	Y	Y	N	N	N	Y	Y	5
Calabria 2024	Y	Y	Y	N	N	N	Y	Y	5
**LABD**									
Coto-Segura 2022	Y	Y	Y	Y	N	N	Y	Y	6
Hali (2) 2022	Y	Y	Y	Y	N	N	Y	Y	6
Han 2022	Y	Y	Y	N	N	N	Y	Y	5
Nahm 2023	Y	Y	Y	Y	N	N	Y	Y	6
**EBA**									
Minakawa 2023	Y	Y	Y	Y	N	N	Y	Y	6
**PV**									
Damiani 2021	Y	Y	Y	N	N	N	Y	Y	5
Solimani 2021	Y	Y	Y	Y	Y	N	Y	Y	7
Agharbi 2022	Y	Y	Y	Y	N	N	Y	Y	6
Akoglu 2022	Y	Y	Y	Y	N	N	Y	Y	6
Aryanian 2022	Y	Y	Y	Y	N	N	Y	Y	6
Avallone 2022 (2)	Y	Y	Y	N	Y	N	Y	Y	6
Calabria 2022	Y	Y	Y	N	N	N	Y	Y	5
Corrá 2022	Y	Y	Y	Y	Y	N	Y	Y	7
Das 2022	Y	Y	Y	N	N	N	Y	Y	5
Hali (1) 2022	Y	Y	Y	N	N	N	Y	Y	5
Hatami 2022	Y	Y	Y	Y	N	N	Y	Y	6
Knecht 2022	Y	Y	Y	N	N	N	Y	Y	5
Koutlas 2022	Y	Y	Y	Y	N	N	Y	Y	6
Norimatsu 2022	Y	Y	Y	Y	N	N	Y	Y	6
Ong 2022	Y	Y	Y	N	N	N	Y	Y	5
Saffarian 2022	Y	Y	Y	Y	N	N	Y	Y	6
Shakoei 2022	Y	Y	Y	Y	N	N	Y	Y	6
Singh 2022	Y	Y	Y	Y	N	N	Y	Y	6
Thongprasom 2022	Y	Y	Y	Y	N	N	Y	Y	6
Saleh 2022	Y	Y	Y	N	N	N	Y	Y	5
Shakoei 2022	Y	Y	Y	Y	N	N	Y	Y	6

Chen 2023	Y	Y	Y	N	N	N	Y	Y	5
Hui 2023	Y	Y	Y	Y	N	N	Y	Y	6
Khalayli 2023	Y	Y	Y	Y	N	N	Y	Y	6
Ligrone 2023	Y	Y	Y	N	N	N	Y	Y	5
Norimatsu 2023	Y	Y	Y	N	N	N	Y	Y	5
**PF**									
Alami 2022	Y	Y	Y	Y	Y	N	Y	Y	7
Corrá 2022	Y	Y	Y	Y	N	N	Y	Y	6
Gui 2022	Y	Y	Y	Y	N	N	Y	Y	6
Hali (1) 2022	Y	Y	Y	Y	N	N	Y	Y	6
Lua 2022	Y	Y	Y	Y	N	N	Y	Y	6
Pourani 2022	Y	Y	Y	Y	N	N	Y	Y	6
Reis 2022	Y	Y	Y	Y	N	N	Y	Y	6
Rouatbi 2022	Y	Y	Y	Y	Y	N	Y	Y	7
Salmi 2022	Y	Y	Y	N	N	N	Y	Y	5
Yildirici 2022	Y	Y	Y	N	Y	N	Y	Y	6
Almasi-Nasrabadi 2023	Y	Y	Y	Y	Y	N	Y	Y	7
Pham 2023	Y	Y	Y	N	N	N	Y	Y	5
**PE**									
Falcinelli 2022	Y	Y	Y	N	N	N	Y	Y	5
**PVeg**									
Gui 2022	Y	Y	Y	Y	N	N	Y	Y	6
**IgA pemphigus**									
Lansang 2023	Y	Y	Y	N	N	N	Y	Y	5
**Pemphigus**									
Almasi-Nasrabadi 2023	Y	Y	Y	N	N	N	Y	Y	5

N, no; Y, yes

Methodological quality and synthesis of case series and case reports by Mural et al.

Selection

Q1: Does the patient(s) represent(s) the whole experience of the investigator (center)?

Ascertainment

Q2: Was the exposure adequately ascertained?

Q3: Was the outcome adequately ascertained?

Causality

Q4: Were other alternative causes that may explain the observation ruled out?

Q5: Was there a challenge/rechallenge phenomenon?

Q6: Was there a dose–response effect?

Q7: Was follow-up long enough for outcomes to occur?

Reporting

Q8: Is the case(s) described with sufficient details to allow other investigators to replicate the research or to allow practitioners make inferences related to their own practice?

Table S3. Quality assessment of observational cohort and cross-sectional studies.

Study	Q1	Q2	Q3	Q4	Q5	Q6	Q7	Q8	Q9	Q10	Q11	Q12	Q13	Q14	Final
**BP**															
Birabaharan 2022	Y	Y	NA	NA	NA	Y	Y	NA	N	U	Y	N	NA	NR	fair
Darrigade 2022	Y	Y	NA	NA	NA	Y	Y	NA	N	U	Y	N	NA	NR	fair
Martora 2022	Y	Y	NA	NA	NA	Y	Y	NA	Y	Y	Y	N	NA	NR	fair
Massip 2022	Y	Y	NA	NA	NA	Y	Y	NA	Y	U	Y	N	NA	NR	fair
McMahon 2022	Y	Y	NA	NA	NA	Y	Y	NA	Y	U	Y	N	NA	NR	fair
Cowan 2023	Y	Y	NA	NA	NA	Y	Y	NA	Y	U	Y	N	NA	NR	fair
Topal 2023	Y	Y	NA	NA	NA	Y	Y	NA	N	U	Y	N	NA	NR	fair
Rasnar 2023	Y	Y	NA	NA	NA	Y	Y	NA	Y	U	Y	N	NA	NR	fair
Diab 2024	Y	Y	NA	NA	NA	Y	Y	NA	N	U	Y	N	NA	NR	fair
**MMP**															
Darrigade 2022	Y	Y	NA	NA	NA	Y	Y	NA	N	U	Y	N	NA	NR	fair
**PV**															
Martora 2022 (2)	Y	Y	NA	NA	NA	Y	Y	NA	Y	Y	Y	N	NA	NR	fair
**Pemphigus**															
Massip 2022	Y	Y	NA	NA	NA	Y	Y	NA	N	U	Y	N	NA	NR	fair
Özgen 2022	Y	Y	NA	NA	NA	Y	Y	NA	Y	Y	Y	N	NA	NR	fair
**PF**															
Weschawalit 2023	Y	Y	NA	NA	NA	Y	Y	NA	Y	U	Y	N	NA	NR	fair
**Not specified**															
Kianfar 2022	Y	Y	NA	NA	NA	Y	Y	NA	N	U	Y	N	NA	NR	fair
Kasperkiewicz 2023	Y	Y	NA	NA	NA	Y	Y	NA	N	U	Y	N	NA	NR	fair

N, no; NA, not applicable; NR, not reported; Y, yes.

NIH quality assessment tool for observational cohort/cross-sectional studies

Questions

Q1: Was the research question or objective in this paper clearly stated?

Q2: Was the study population clearly specified and defined?

Q3: Was the participation rate of eligible persons at least 50%?

Q4: Were all the subjects selected or recruited from the same or similar populations (including the same time period)? Were the inclusion and exclusion criteria for being in the study prespecified and applied uniformly to all participants?

Q5: Was a sample size justification, power description, or variance and effect estimated provided?

Q6: For the analysis in this paper, were the exposure(s) of interest measured prior to the outcome(s) being measured?

Q7: Was the timeframe sufficient so that one could reasonably expect to see an association between exposure and outcome if it existed?

Q8: For exposures that can vary in amount or level, did the study examine different levels of the exposure as related to the outcome (e.g., categories of exposure, or exposure measured as continuous variable)?

Q9: Were the exposure measures (independent variables) clearly defined, valid, reliable, and implemented consistently across all study participants?

Q10: Was the exposure(s) assessed more than once over time?

Q11: Were the outcome measures (dependent variables) clearly defined, valid, reliable, and implemented consistently across all study participants?

Q12: Were the outcome assessors blinded to the exposure status of participants?

Q13: Was loss to follow-up after baseline 20% or less?

Q14: Were key potential confounding variables measured and adjusted statistically for their impact on the relationship between exposure(s) and outcome(s)?

Final rating

Quality was rated as poor (0–4 out of 14 questions), fair (5–10 out of 14 questions), or good (11–14 out of 14 questions)

Table S4. The assessment of Naranjo score for cases of new onset autoimmune bullous dermatosis.

Study	Case No.	Q1	Q2	Q3	Q4	Q5	Q6	Q7	Q8	Q9	Q10	Final score
BP												
Khalid 2021	1	0	2	0	2	2	0	0	0	1	0	7
Nakamura 2021	1	0	2	0	0	2	0	0	0	0	0	4
Pérez-López 2021	1	0	2	0	2	0	0	0	0	1	0	5
Tomayko 2021	1	0	2	0	0	0	0	0	0	0	0	2
	2	0	2	0	0	0	0	0	0	0	0	2
	3	0	2	0	0	0	0	0	0	0	0	2
	4	0	2	0	0	0	0	0	0	0	0	2
	5	0	2	0	-1	0	0	0	0	0	0	1
	6	0	2	0	0	0	0	0	0	0	0	2
	7	0	2	0	0	0	0	0	0	0	0	2
	8	0	2	0	0	0	0	0	0	0	0	2
	9	0	2	0	0	0	0	0	0	0	0	2
	10	0	2	0	2	0	0	0	0	1	0	5
	11	0	2	0	-1	0	0	0	0	0	0	1
	12	0	2	0	0	0	0	0	0	0	0	2
Afacan 2022	1	0	2	0	0	0	0	0	0	0	0	2
	2	0	2	0	0	0	0	0	0	0	0	2
	3	0	2	0	0	0	0	0	0	0	0	2
	4	0	2	0	0	0	0	0	0	0	0	2
Agharbi 2022 (1)	1	0	2	0	0	2	0	0	0	0	0	4
Alshammari 2022	1	0	2	0	0	0	0	0	0	0	0	2
Avallone 2022	1	0	2	0	0	2	0	0	0	0	0	4
Bailly-Caille 2022	1	0	2	0	0	2	0	0	0	0	0	4
Bardazzi 2022	1	0	2	0	0	0	0	0	0	0	0	2
	2	0	2	0	0	0	0	0	0	0	0	2
Birabaharan 2022	1	0	2	0	0	0	0	0	0	0	0	2
Bostan 2022	1	0	2	0	2	-1	0	0	0	1	0	4
Coto-Segura 2022	1	0	2	0	0	0	0	0	0	0	0	2
	2	0	2	0	0	0	0	0	0	0	0	2
	3	0	2	0	0	0	0	0	0	0	0	2
Daines 2022	1	0	2	0	0	2	0	0	0	0	0	4
Darrigade 2022	1	0	2	0	0	0	0	0	0	0	0	2
Dell'Antonia 2022	1	0	2	0	2	2	0	0	0	1	0	7
Desai 2022	1	0	2	0	2	2	0	0	0	1	0	7
Fu 2022	1	0	2	0	0	0	0	0	0	0	0	2
Gambichler 2022	1	0	2	0	2	2	0	0	0	1	0	7
	2	0	2	0	0	2	0	0	0	0	0	4

Guo 2022	1	0	2	0	2	2	0	0	0	1	0	7
	2	0	2	0	0	2	0	0	0	0	0	4
Hali (1) 2022	1	0	2	0	0	2	0	0	0	0	0	4
	2	0	2	0	0	2	0	0	0	0	0	4
	3	0	2	0	2	2	0	0	0	1	0	7
Hung 2022	1	0	2	0	0	0	0	0	0	0	0	2
Larson 2022	1	0	2	0	2	2	0	0	0	1	0	7
	2	0	2	0	0	2	0	0	0	0	0	4
McMahon 2022	1	0	2	0	0	0	0	0	0	0	0	2
Maronese 2022 (1)	1	0	2	0	0	0	0	0	0	0	0	2
	2	0	2	0	0	0	0	0	0	0	0	2
	3	0	2	0	0	0	0	0	0	0	0	2
	4	0	2	0	0	0	0	0	0	0	0	2
	5	0	2	0	0	0	0	0	0	0	0	2
	6	0	2	0	0	0	0	0	0	0	0	2
	7	0	2	0	0	0	0	0	0	0	0	2
	8	0	2	0	0	0	0	0	0	0	0	2
	9	0	2	0	0	0	0	0	0	0	0	2
	10	0	2	0	0	0	0	0	0	0	0	2
	11	0	2	0	0	0	0	0	0	0	0	2
	12	0	2	0	0	0	0	0	0	0	0	2
	13	0	2	0	0	0	0	0	0	0	0	2
	14	0	2	0	0	0	0	0	0	0	0	2
	15	0	2	0	0	0	0	0	0	0	0	2
	16	0	2	0	0	0	0	0	0	0	0	2
	17	0	2	0	0	0	0	0	0	0	0	2
	18	0	2	0	0	0	0	0	0	0	0	2
	19	0	2	0	0	0	0	0	0	0	0	2
	20	0	2	0	0	0	0	0	0	0	0	2
	21	0	2	0	0	0	0	0	0	0	0	2
Maronese 2022 (2)	1	0	2	0	0	-1	0	0	0	0	0	1
	2	0	2	0	0	-1	0	0	0	0	0	1
	3	0	2	0	0	-1	0	0	0	0	0	1
Nakahara 2022	1	0	2	0	0	-1	0	0	0	0	0	1
Nida 2022	1	0	2	0	0	0	0	0	0	0	0	2
Pauluzzi 2022	1	0	2	0	0	0	0	0	0	0	0	2
Russo 2022	1	0	2	0	0	-1	0	0	0	0	0	1
Savoldy 2022	1	0	2	0	2	2	0	0	0	1	0	7
Schmidt 2022	1	0	2	0	2	2	0	0	0	1	0	7
Shakoei 2022	1	0	2	0	0	2	0	0	0	0	0	4
	2	0	2	0	0	2	0	0	0	0	0	4

Shanshal 2022	1	0	2	0	2	2	0	0	0	1	0	7
Wan 2022	1	0	2	0	0	2	0	0	0	0	0	4
	2	0	2	0	2	2	0	0	0	1	0	7
Young 2022	1	0	2	0	2	2	0	0	0	1	0	7
Zhang 2022	1	0	2	0	0	0	0	0	0	0	0	2
	2	0	2	0	0	0	0	0	0	0	0	2
Baffa 2023	1	0	2	0	0	2	0	0	0	0	0	4
Cowan 2023	1	0	2	0	0	0	0	0	0	0	0	2
	2	0	2	0	0	0	0	0	0	0	0	2
	3	0	2	0	0	0	0	0	0	0	0	2
	4	0	2	0	0	0	0	0	0	0	0	2
Dawoud 2023	1	0	2	0	0	0	0	0	0	0	0	2
	2	0	2	0	0	0	0	0	0	0	0	2
Hsieh 2023	1	0	2	0	0	2	0	0	0	0	0	4
Mulianto 2023	1	0	2	0	0	2	0	0	0	0	0	4
Sun 2023	1	0	2	0	0	2	0	0	0	0	0	4
Topal 2023	1	0	2	0	0	0	0	0	0	0	0	2
Ustün 2023	1	0	2	0	0	2	0	0	0	0	0	4
Diab 2024	1	0	2	0	0	0	0	0	0	0	0	2
	2	0	2	0	0	0	0	0	0	0	0	2
Yamamoto 2024	1	0	2	0	0	0	0	0	0	0	0	2
PGes												
Mustin 2023	1	0	2	0	0	-1	0	0	0	0	0	1
MMP												
Darrigade 2022	1	0	2	0	0	0	0	0	0	0	0	2
Rungraungrayabkul 2023	1	0	2	0	0	2	0	0	0	0	0	4
Calabria 2024	1	0	2	0	0	0	0	0	0	0	0	2
LABD												
Coto-Segura 2022	1	0	2	0	0	2	0	0	0	0	0	4
Hali (2) 2022	1	0	2	0	0	2	0	0	0	0	0	4
Han 2022	1	0	2	0	0	2	0	0	0	0	0	4
Nahm 2023	1	0	2	0	0	2	0	0	0	0	0	4
PV												
Solimani 2021	1	0	2	0	2	2	0	0	0	1	0	7
Agharbi 2022 (2)	1	0	2	0	0	2	0	0	0	0	0	4
Akoglu 2022	1	0	2	0	0	2	0	0	0	0	0	4
Aryanian 2022	1	0	2	0	0	2	0	0	0	0	0	4
Calabria 2022	1	0	2	0	0	0	0	0	0	0	0	2
Corrá 2022	1	0	2	0	0	2	0	0	0	0	0	4
	2	0	2	0	0	2	0	0	0	0	0	4
	3	0	2	0	2	2	0	0	0	1	0	7

Das 2022	1	0	2	0	0	0	0	0	0	0	0	2
Hali (2) 2022	1	0	2	0	0	0	0	0	0	0	0	2
Hatami 2022	1	0	2	0	0	2	0	0	0	0	0	4
Knecht 2022	1	0	2	0	0	0	0	0	0	0	0	2
Koutlas 2022	1	0	2	0	0	2	0	0	0	0	0	4
Norimatsu 2022	1	0	2	0	0	2	0	0	0	0	0	4
Saffarian 2022	1	0	2	0	0	2	0	0	0	0	0	4
Shakoei 2022	1	0	2	0	0	2	0	0	0	0	0	4
Singh 2022	1	0	2	0	0	2	0	0	0	0	0	4
Thongprasom 2022	1	0	2	0	0	2	0	0	0	0	0	4
Cowan 2023	1	0	2	0	0	0	0	0	0	0	0	2
Hui 2023	1	0	2	0	0	2	0	0	0	0	0	4
Khalayli 2023	1	0	2	0	0	2	0	0	0	0	0	4
Norimatsu 2023	1	0	2	0	0	0	0	0	0	0	0	2
Diab 2024	1	0	2	0	0	0	0	0	0	0	0	2
PF												
Alami 2022	1	0	2	0	2	2	0	0	0	1	0	7
Corrá 2022	1	0	2	0	0	2	0	0	0	0	0	4
	2	0	2	0	-1	2	0	0	0	0	0	3
Gui 2022	1	0	2	0	0	2	0	0	0	0	0	4
Hali (2) 2022	1	0	2	0	0	2	0	0	0	0	0	4
Lua 2022	1	0	2	0	0	2	0	0	0	0	0	4
Pourani 2022	1	0	2	0	0	2	0	0	0	0	0	4
Reis 2022	1	0	2	0	0	2	0	0	0	0	0	4
Rouatbi 2022	1	0	2	0	0	2	0	0	0	0	0	4
	2	0	2	0	2	2	0	0	0	1	0	7
Yildirici 2022	1	0	2	0	2	0	0	0	0	1	0	5
Almasi-Nasrabadi 2023	1	0	2	0	2	2	0	0	0	1	0	7
Pham 2023	1	0	2	0	0	2	0	0	0	0	0	4
	2	0	2	0	0	2	0	0	0	0	0	4
Weschawalit 2023	1	0	2	0	0	0	0	0	0	0	0	2
Diab 2024	1	0	2	0	0	0	0	0	0	0	0	2
PE												
Falcinelli 2022	1	0	2	0	0	0	0	0	0	0	0	2
PVeg												
Gui 2022	1	0	2	0	0	2	0	0	0	0	0	4
IgA pemphigus												
Lansang 2023	1	0	2	0	0	2	0	0	0	0	0	4
Not specified												
Kianfar 2022	1	0	2	0	0	0	0	0	0	0	0	2

Q1: Are there previous conclusive reports on this reaction? (+1 yes, 0 no, 0 do not know)

Q2: Did the adverse event appear after the suspected drug was administered? (+2 yes, -1 no, 0 do not know)

Q3: Did the adverse event improve when the drug was discontinued or a specific antagonist was administered? (+1 yes, 0 no, 0 do not know)

Q4: Did the adverse event reappear when the drug was readministered? (+2 yes, -1 no, 0 do not know)

Q5: Are there alternative causes that could on their own have caused the reaction? (-1 yes, +2 no, 0 do not know)

Q6: Did the reaction reappear when a placebo was given? (-1 yes, +1 no, 0 do not know)

Q7: Was the drug detected in blood or other fluids in concentrations known to be toxic? (+1 yes, 0 no, 0 do not know)

Q8: Was the reaction more severe when the dose was increased or less severe when the dose was decreased? (+1 yes, 0 no, 0 do not know)

Q9: Did the patient have a similar reaction to the same or similar drugs in any previous exposure? (+1 yes, 0 no, 0 do not know)

Q10: Was the adverse event confirmed by any objective evidence? (+1 yes, 0 no, 0 do not know)

Table S5. The assessment of Naranjo score for cases of exacerbation of autoimmune bullous dermatosis.

Study	Case No.	Q1	Q2	Q3	Q4	Q5	Q6	Q7	Q8	Q9	Q10	Final score
BP												
Damiani 2021	1	0	2	0	-1	0	0	0	0	0	0	1
	2	0	2	0	2	0	0	0	0	1	0	5
	3	0	2	0	-1	0	0	0	0	0	0	1
Tomayko 2021	1	0	2	0	0	0	0	0	0	0	0	2
Afacan 2022	1	0	2	0	0	0	0	0	0	0	0	2
	2	0	2	0	0	0	0	0	0	0	0	2
	3	0	2	0	0	0	0	0	0	0	0	2
Bardazzi 2022	1	0	2	0	0	0	0	0	0	0	0	2
	2	0	2	0	0	0	0	0	0	0	0	2
Happaerts 2022	1	0	2	0	0	2	0	0	0	0	0	4
Juay 2022	1	0	2	0	0	2	0	0	0	0	0	4
Martora 2022	1	0	2	0	-1	0	0	0	0	0	0	1
Massip 2022	1	0	2	0	0	0	0	0	0	0	0	2
Cowan 2023	1	0	2	0	0	0	0	0	0	0	0	2
	2	0	2	0	0	0	0	0	0	0	0	2
	3	0	2	0	0	0	0	0	0	0	0	2
Rasner 2023	1	0	2	0	0	2	0	0	0	0	0	4
	2	0	2	0	0	2	0	0	0	0	0	4

EBA												
Minakawa 2023	1	0	2	0	0	2	0	0	0	0	0	4
PV												
Damiani 2021	1	0	2	0	-1	0	0	0	0	0	0	1
	2	0	2	0	-1	0	0	0	0	0	0	1
Akoglu 2022	1	0	2	0	2	2	0	0	0	1	0	7
	2	0	2	0	0	2	0	0	0	0	0	4
Avallone 2022	1	0	2	0	2	0	0	0	0	1	0	5
Hatami 2022	1	0	2	0	0	0	0	0	0	0	0	2
Martora 2022 (2)	1	0	2	0	0	0	0	0	0	0	0	2
Ong 2022	1	0	2	0	0	0	0	0	0	0	0	2
Saleh 2022	1	0	2	0	0	0	0	0	0	0	0	2
Shakoei 2022	1	0	2	0	0	2	0	0	0	0	0	4
Chen 2023	1	0	2	0	0	0	0	0	0	0	0	2
Cowan 2023	1	0	2	0	0	0	0	0	0	0	0	2
	2	0	2	0	0	0	0	0	0	0	0	2
Ligrone 2023	1	0	2	0	0	0	0	0	0	0	0	2
PF												
Salmi 2022	1	0	2	0	0	0	0	0	0	0	0	2
Rasner 2023	1	0	2	0	0	0	0	0	0	0	0	2
Pemphigus												
Massip 2022	1	0	2	0	0	0	0	0	0	0	0	2
Özgen 2022	1	0	2	0	0	0	0	0	0	0	0	2
Not specified												
Kasperkiewicz 2023	1	0	2	0	0	0	0	0	0	0	0	2
Kianfar 2022	1	0	2	0	0	0	0	0	0	0	0	2

Q1: Are there previous conclusive reports on this reaction? (+1 yes, 0 no, 0 do not know)

Q2: Did the adverse event appear after the suspected drug was administered? (+2 yes, -1 no, 0 do not know)

Q3: Did the adverse event improve when the drug was discontinued or a specific antagonist was administered? (+1 yes, 0 no, 0 do not know)

Q4: Did the adverse event reappear when the drug was readministered? (+2 yes, -1 no, 0 do not know)

Q5: Are there alternative causes that could on their own have caused the reaction? (-1 yes, +2 no, 0 do not know)

Q6: Did the reaction reappear when a placebo was given? (-1 yes, +1 no, 0 do not know)

Q7: Was the drug detected in blood or other fluids in concentrations known to be toxic? (+1 yes, 0 no, 0 do not know)

Q8: Was the reaction more severe when the dose was increased or less severe when the dose was decreased? (+1 yes, 0 no, 0 do not know)

Q9: Did the patient have a similar reaction to the same or similar drugs in any previous exposure? (+1 yes, 0 no, 0 do not know)

Q10: Was the adverse event confirmed by any objective evidence? (+1 yes, 0 no, 0 do not know)
